# Official Preparations of Uncertain Strength

**Published:** 1890-02

**Authors:** 


F. E. DANIEL, M. D., EDITOR AND PUBLISHER.

THIS JOURNAL, by unanimous vote of the members, was made the Official Organ of the Austin District Medical Society.

COLLABORATORS:

*Dr. R. M. Swearingen, Austin.*

*Prof. B. E. Hadra, M. D., Galveston.*

*Prof. Geo. Cupples, M. D., San Antonio.*

*Dr. T. C. Osborn, Cleburne, Texas.*

*Dr E. J. Doering, Chicago.*

*Dr. E. J. Beall, Fort Worth.*

*Dr Odo Betz, Germany.*

*Dr. J. W. McLaughlin, Austin.*

*Dr. T. J. Tyner, Austin.*

*Prof. J. F. Y. Paine, M. D., Galveston.*

*Dr. R. H. L. Bibb, Mexico.*

*Dr. T. J. Bennett, Austin.*

*Dr. Bat Smith, Wharton.*

*Dr. E. Meierhof, New York.*

*Prof. W. B. Rogers, M. D., Memphis, Tenn.*

### OFFICIAL PREPARATIONS OF UNCERTAIN STRENGTH.

The time is rapidly approaching, and is near at hand when a committee composed of delegates from, and representatives of all chartered medical and pharmaceutical bodies in the United States will meet in the national capital to revise the United States pharmacopœia.

It behooves all thinking medical men to take an interest in this matter; for,—as a valued contemporary says,—"progress in pharmacy is progress in medicine;" and the physician is beset with no greater evil than that which he encounters daily in the variable and uncertain effects of official remedies, because of their variable and uncertain strength.

The pharmacopœia is the standard and guide in the preparation of the drugs and medicines which he daily uses in his practice; and a physician's failure or success as a practitioner is largely influenced by the means he employs in the treatment of disease. Experience has demonstrated many of the methods of preparation heretofore practiced by pharmacists in accordance with directions given in this work, to be defective and unsatisfactory; and every decade there is a revision; those remedies and preparations which have been found to be worthless have been thrown out; and others added, as the investigation of pharmacists, or observation and experience of physicians have developed new drugs, or better modes of preparation. It is admitted on all hands that there are many defects in the present system; this has been abundantly demonstrated in the experience of the profession in the ten years that have elapsed since the last over-hauling.

But there are none more glaring and palpable than in the directions given for making tinctures and fluid-extracts. When it is considered that the amount of "virtue" in a specimen or package of a crude drug, whether leaves, root, seeds or bark, depends so largely on the source whence octained, the mode of curing, packing, shipping; the age, surroundings as to heat or moisture, exposure or protection etc.; for in a very large number of drugs, the active principle is of a volatile or easily decomposable nature, it can be readily seen how absurd are the directions given in the United States pharmacopœia which require that one gramme of drug, regardless of its quality, shall make one cubic centimetre of fluid extract. It is about as definite as if one should say, "take a piece about the size of a lump of chalk."

The necessity of a standard as to the virtue or active principle in the crude drug in the preparation of fluid extracts or tinctures is clearly manifest in the preparation of the toxic drugs; the strength of the extract depends, of course, upon the the strength or per cent. of the alkaloid or oil (in many instances) resident in the crude material; and in case of poisonous drugs like belladonna or aconite, it is of the highest importance that the therapeutic value of these preparations should be as definite as pharmaceutical skill can make them. All practitioners will recall the disapointment and vexation they have experienced, when, perhaps in some critical case, they have cautiously administered, say, a tincture of aconite, and gradually, drop by drop, increased the dose till the limit of safety in their judgment has been reached, and observed no physiological effects on the patient. This fact is so well know that it is useless to argue the point.

But who is to fix this standard? It seems to us that the committee who have the authority to revise the pharmacopœia should be empowered by and at the expense of the government, to fix the standard of all assayable preparations. A suitable commission should be appointed for the purpose of inspecting the preparations of the market, allowing a reasonable margin for the variation in the quality of the finished product; an assayer for each district might be appointed, and in this way the difficulty complained of could, in a great measure, be remedied. There is, however, no fund available for this purpose at present.

But is there not a better plan? Let the standard strength of extracts and tinctures be fixed, and let them be assayed, and required to conform to this standard when put upon the market.

Messrs. Parke, Davis & Co., the intelligent and progressive manufacturers, of Detroit, and whom we regard as the physician's friend and ally, long ago saw the glaring defect we have just alluded to, and on their own hook put on the market a number of preparations of standard strength, which they call "Normal Liquids." As these manufacturers are in direct contact with physicians, and reach them through the local pharmacists, the medical profession were—dissatisfied with the uncertain strength of the U. S. P. tinctures and fluid extracts—easily and quickly induced to try them. The results were uniform and satisfactory, and a big demand and extensive sale was the result. "Normal Liquids" soon took the place, to a large extent, of the "official" preparations, both on the shelf of the apothecary and in the doctor's "saddle-bags."

Why not adopt "Normal Liquids" as a name, and officially require the same standards? Parke, Davis & Co., nor any other house, have any interest in the name "Normal Liquids." It is not patented; nor protected by trade-mark. They only suggest it as *indicative of an ascertained and uniform strength*—a standard —the very thing that is wanted; and even should the adoption into the U. S. P. of the formula used by them redound largely, or even exclusively to their profit commercially, we know of none more worthy to receive, nor better entitled to such results. But were this done, all chemists would have an equal right to manufacture "Normal Liquids" by the methods communicated, and

